# A 20 bp Duplication in Exon 2 of the Aristaless-Like Homeobox 4 Gene (*ALX4*) Is the Candidate Causative Mutation for Tibial Hemimelia Syndrome in Galloway Cattle

**DOI:** 10.1371/journal.pone.0129208

**Published:** 2015-06-15

**Authors:** Bertram Brenig, Ekkehard Schütz, Michael Hardt, Petra Scheuermann, Markus Freick

**Affiliations:** 1 Institute of Veterinary Medicine, Georg-August-University of Göttingen, 37077 Göttingen, Germany; 2 Landesuntersuchungsanstalt für das Gesundheits- und Veterinärwesen Sachsen, 04158 Leipzig, Germany; 3 Veterinary Practice Zettlitz, Straße der Jugend 68, 09306 Zettlitz, Germany; Wageningen UR Livestock Research, NETHERLANDS

## Abstract

Aristaless-like homeobox 4 (*ALX4*) gene is an important transcription regulator in skull and limb development. In humans and mice *ALX4* mutations or loss of function result in a number of skeletal and organ malformations, including polydactyly, tibial hemimelia, omphalocele, biparietal foramina, impaired mammary epithelial morphogenesis, alopecia, coronal craniosynostosis, hypertelorism, depressed nasal bridge and ridge, bifid nasal tip, hypogonadism, and body agenesis. Here we show that a complex skeletal malformation of the hind limb in Galloway cattle together with other developmental anomalies is a recessive autosomal disorder most likely caused by a duplication of 20 bp in exon 2 of the bovine *ALX4* gene. A second duplication of 34 bp in exon 4 of the same gene has no known effect, although both duplications result in a frameshift and premature stop codon leading to a truncated protein. Genotyping of 1,688 Black/Red/Belted/Riggit Galloway (GA) and 289 White Galloway (WGA) cattle showed that the duplication in exon 2 has allele frequencies of 1% in GA and 6% in WGA and the duplication in exon 4 has frequencies of 23% in GA and 38% in WGA. Both duplications were not detected in 876 randomly selected German Holstein Friesian and 86 cattle of 21 other breeds. Hence, we have identified a candidate causative mutation for tibial hemimelia syndrome in Galloway cattle and selection against this mutation can be used to eliminate the mutant allele from the breed.

## Introduction

Vertebrate limb development is a complex process regulated by two signalling centres, the apical ectodermal ridge (AER) and the zone of polarizing activity (ZPA) [1, 2]. Within these centres, several genes coordinate limb growth along the anteroposterior (AP), dorsoventral and proximodistal axes. Once the limb has formed from the shoulder and/or pelvic girdle to the distal tip, the mesenchymal cells begin to condense, differentiate into cartilage, and finally into bone. Although the detailed interactions of many factors involved in this process are still unknown, most of the important genes have been identified [2]. Besides fibroblast growth factors expressed in the AER, sonic hedgehog (SHH) is important in the ZPA for AP patterning. However, AP patterning is controlled by additional factors that are also SHH-independent. Signalling of *GLI3* expressed in the anterior mesenchym for example is responsible for pre-patterning of the limb bud before SHH signalling [2].

Aristaless-like homeobox 4 (ALX4) is another transcription regulator involved in skull and limb development and interacts with a plethora of other transcription regulators, *e*.*g*. SHH, lymphoid enhancer-binding factor 1 (LEF1), exostosin 2 (EXT2), sex determining region Y-box 10 (SOX10), ALX homeobox 1 (ALX1), and GLI family zinc finger 3 (GLI3) [3–7]. Expression of *ALX4* has been detected in osteoblast precursors of most bones, the dermal papilla of hair and whisker follicles, the dental papilla of teeth, and a subset of mesenchymal cells in pubescent mammary glands [8].

In limb development, *ALX4* expression in the anterior margin limb bud is under the control of GLI3 and SHH. Within this cascade, disruption of *ALX4* results in an anterior ectopic expression of SHH and the formation of extra digits as shown in Alx4^-/-^ mice [9, 10]. Due to its restricted expression at sites of epithelial-mesenchymal interactions, ALX4 is also important in calvarial bone development. In the congenital hydrocephalus mutant mouse, downregulation of *ALX4* expression as a result of forkhead/winged helix transcription factor (Foxc1) loss of function disrupts the progression of osteogenesis [11]. Mutations in the human *ALX4* gene have been shown to be causative for a number of similar skull and limb defects [12–17].

Different congenital skeletal malformations have been reported in livestock, however, only a few have been clarified on a molecular level so far, *e*. *g*. brachyspina, complex vertebral malformation, and syndactyly [18–24]. Cases of tibial hemimelia (TH) have been reported in Galloway, Bunaji, and Shorthorn cattle [25–28]. The first report on TH in cattle was published in 1951 in the Scottish Galloway breed [28]. Affected calves were either stillborn or died shortly after birth and showed multiple congenital skeletal deformaties, including shortened or absent tibia, abdominal hernia, cryptorchidism, failed Müllerian duct development, hirsutism, and improper neural tube closure, resulting in meningocele [29]. In the early 1970´s further cases of TH in Galloway cattle were reported in Germany and the US [26]. In these studies 12 deformed Galloway calves have been analyzed with moderate to severe internal hydrocephalus, meningoencephalocele, ventral abdominal hernia, and bilateral agenesis of the patella and tibia [30].

In Holstein Friesian, a complete absence of the thoracic limbs (amelia) has been associated with chromosomal instabilities without an inherited background. Hence, it can be speculated that the etiology of limb malformations might be heterogenous including hereditary as well as environmental factors [31]. In humans, congenital aplasia and dysplasia of the tibia with intact fibula has been reported in the late 1970´s and classified into four types of deformation ranging from a total absence of the tibia including a hypoplastic distal femoral epiphysis, presence of proximal or distal parts of the tibia to diastasis [32]. In several cases, additional visceral and skeletal deformities of other limbs, *i*. *e*. femur, foot, double fibula, were also found [32]. Although, in bovine syndactyly of German Holstein, German Fleckvieh and crossbreeds, *ALX4* was excluded as a candidate gene [33], its envolvement in other skeletal malformations remains elusive. Due to its key role in limb development, we hypothesized that *ALX4* is a candidate gene for tibial hemimelia syndrome in Galloway cattle.

## Materials and Methods

### Blood, tissue and DNA samples

A total of 1688 DNA samples of the most common Galloway varieties (Black/Red/Belted/Riggit Galloway: GA), and 289 White Galloway (WGA) were randomly selected from the DNA depository at the Institute of Veterinary Medicine. In addition, 876 German Holstein Friesian (HF) and 86 DNA samples of 21 different cattle breeds, *i*.*e*. Aberdeen Angus (2), Aubrac (1), Blonde d´Aquitaine (5), Brown Swiss (3), Charolais (9), Chianina (7), German Angus (5), German Black Pied cattle (2), German Simmental (5), Glanrind (5), German Yellow cattle (1), Hereford (4), Scottish Highland (3), Limousin (4), Piemonteser (7), Red Holstein (8), German Red Highlander (1), Angler (3), Welsh Black (2), Belgian Blue (2), and White Park (7) were included.

Tissue samples of two TH affected Galloway cattle (V.1, V.2) and hair samples of their relatives were provided by K. Kipping (Rüx/Germany). Blood samples were drawn by Dr. M. Freick as part of routine diagnostic procedures (parentage control, epidemiological testing) with informed owner consent, therefore the study was exempt of ethical approval according to the German regulations.

A total of 15 DNA samples were available for the analysis. DNA from blood samples was extracted using a salting out procedure [34] or the MagNA Pure LC DNA Isolation Kit I (Roche Diagnostics). For the isolation of DNA from tissue samples the DNeasy Blood and Tissue Kit (Qiagen) or the MagNA Pure LC DNA Isolation Kit II (Roche Diagnostics) was used according to the manufacturer´s protocols.

### *ALX4* sequence, PCR primers, and analysis of mutations

The bovine *ALX4* gene is located on BTA15q28-q29 between positions 74,452,084–74,486,658 Mb and harbours 4 exons coding for a protein of 397 amino acids [33]. Due to the fact that the *ALX4* gene has not yet been correctly annotated and larger gaps exist in intron 1 and 2, it is not possible to give exact distances. As reference the bovine *ALX4* DNA sequence deposited with accession number NC_007313 at NCBI (Btau_4.6.1) and AC_000172 (Bos_taurus_UMD3.1) was used. Numbering of positions refers to AC_000172 (Bos_taurus_UMD3.1) and complies with the Human Genome Variation Society (HGVS) nomenclature [35]. For comparison of sequences, exons and adjacent intronic regions were amplified using PCR primers shown in Table 1. PCR primers were designed using the online software tool Primer3 [36].

**Table 1:** PCR primers for bovine *ALX4* gene amplification and mutation analysis.

Primer	5´- 3´ ^a)^	Position ^b)^	T_a_ (°C) ^c)^	Product size (bp)
*Exon 1 primers*				
Alx4ex1fwd	CCTCCTGGCCTCTCCTAACT	75187092..75187111	52	598
Alx4ex1rev	CAGCAAGTTGATCGCGTTT	75186514..75186532		
*Exon 2 primers*				
BT_ALX4_Ex2_fwd	GGGATGGGGAGACAGACTAG	75178748..75178767	63	229
BT_ALX4_Ex2_rev	ACCCAGAGCTCTTGATGTCC	75178539..75178558		
ALX4_Ex2fwd	TCCCTCCTACCTCTCGGGC	75154752..75154770	55	443
ALX4_Ex2rev	CCTGTCTCGGGCCACTG	75154328..75154344		
*Exon 3 primers*				
ALX4_Ex3fwd	GATTCTGCCGTAGTCTGTGG	75364265..75364284	62	786
ALX4_Ex3rev	CTTCAGCATTCCTCGGTTC	75363499..75363517		
*Exon 4 primers*				
ALX4_Ex4fwd	AAAGCCTCCCAGGTAAACAC	75361464..75361483	60	611
ALX4_Ex4rev	GAAAGTGCTGAGGGTCAGG	75360873..75360891		
bALX4_rev	GCCAAGACGGTGCTCAGGC	75179672..75179690	60	306
bALX4_del	ATCCTGTGCGACCCCCTCCC	75179957..75179976		
*Exon 2 (FRET primers)*				
bALX4_RNA_Ex2F_neu	GAAGACCCACTACCCCGATG	75154452..75154471	60	144
ALX4_Ex2rev	CCTGTCTCGGGCCACTG	75154328..75154344		
bALX4_Ex2_Probe	CGTGACCTCACCGAGGCCC(Flc)			
bALX4_Ex2_Anchor	(Cy5)TGCAGGTCAGTGAGGGTGCCAGGGAAG(Pho)	75154374..75154400		
*Exon 4 (FRET primers)*				
bALX4_RNA_Ex4F	CAGAACCCGTCCTGGATC	75361324..75361342	67	136
bALX4_RNA_Ex4R	AGTCGGTGACGCCGCT	75361207..75361222		
bALX4Ex4_Probe	CCCGGTGCGTGCCGGCCTGTGT(Flc)			
bALX4Ex4_Anchor	(Cy5)GCCCTGTGACCCGGTGCCCGCCTGCATGTC(Pho)	75361254..75361283		

*Note*. a) Flc: Fluorescein, Cy5: Cyanine 5, Pho: Phosphorylation

b) Positions refer to AC_000172 (Bos_taurus_UMD3.1)

c) T_a_ = annealing temperature

For high throughput genotyping of the duplications in exon 2 and 4 fluorescence resonance energy transfer (FRET) assays were developed [37, 38]. Analysis of exon 2 was done in a total volume of 5 μl, 0.3 U *Taq* DNA polymerase (Roche Diagnostics GmbH), 20 pmol dNTPs each (Roche Diagnostics GmbH), 0.6 pmol bALX4_RNA_Ex2F_neu, 2 pmol ALX4_Ex2rev, 4 pmol bALX4_Ex2_Probe, 4 pmol bALX4_Ex2_Anchor, 1 x PCR buffer (incl. MgCl_2_, Roche Diagnostics GmbH), 1 x Q-Solution, 20 ng template DNA were mixed and cycled in a LightCycler 480 (Roche Diagnostics GmbH) using filter set 483–670 nm (Table 1). Amplification was done with an initial denaturation at 95°C, 5 min, followed by 30–35 cycles at 95°C, 30 sec, 60°C, 1 min, 72°C 30 sec and a final elongation at 72°C for 5 min. Melting was done at 95°C, 30 sec (4.4°C/sec), 50°C, 1 sec (2.2°C/sec), 80°C acquisition mode continuous (0.29°C/sec), and 50°C, 30 sec (2.2°C/sec).

Analysis of exon 4 was done in a total volume of 10 μl, 0.6 U *Taq* DNA polymerase (Roche Diagnostics GmbH), 40 pmol dNTPs each (Roche Diagnostics GmbH), 4 pmol bALX4_RNA_Ex4F, 12 pmol bALX4_RNA_Ex4R, 4 pmol bALX4_Ex4_Probe, 20 pmol bALX4_Ex4_Anchor, 1 x PCR buffer (incl. MgCl_2_, Roche Diagnostics GmbH), 1 x Q-Solution, 20 ng template DNA were mixed and cycled in a LightCycler 480 (Roche Diagnostics GmbH) using filter set 483–670 nm (Table 1). Amplification was done with an initial denaturation at 95°C, 5 min, followed by 45–50 cycles at 95°C, 30 sec, 67°C, 1 min, 72°C 30 sec and a final elongation at 72°C for 5 min. Melting was done at 95°C, 30 sec (4.4°C/sec), 50°C, 1 sec (2.2°C/sec), 90°C acquisition mode continuous (0.29°C/sec), and 50°C, 30 sec (2.2°C/sec).

### DNA sequencing and analysis

PCR products were purified using the ExoSAP-IT PCR Product Cleanup (USB) and sequenced with the BigDye Terminator v3.1 Cycle Sequencing Kit (Applied Biosystems) on an ABI PRISM 3130xl Genetic Analyzer (Life Technologies) according to the manufacturers´ protocols. Sequencing primers are listed in Table 1 and are the same as used for PCR. For sequence comparison and identification of sequence variations seven animals of the cattle family including the two affected calves, i.e. 573, 954, 955, 957, 958, V.1, and V.2, were used.

Raw DNA sequence data were imported into Sequencher 5.2.3 (Build 12903) (Gene Codes) and manually checked for ambiguities using NC_007313 (Btau 4.6.1) and AC_000172 (UMD 3.1) as reference sequence.

For *in silico* prediction of splice site mutation effects NNSPLICE 0.9 and Human Splicing Finder was used [39, 40].

## Results

### Clinical and pathological analysis

**Fig 1:**
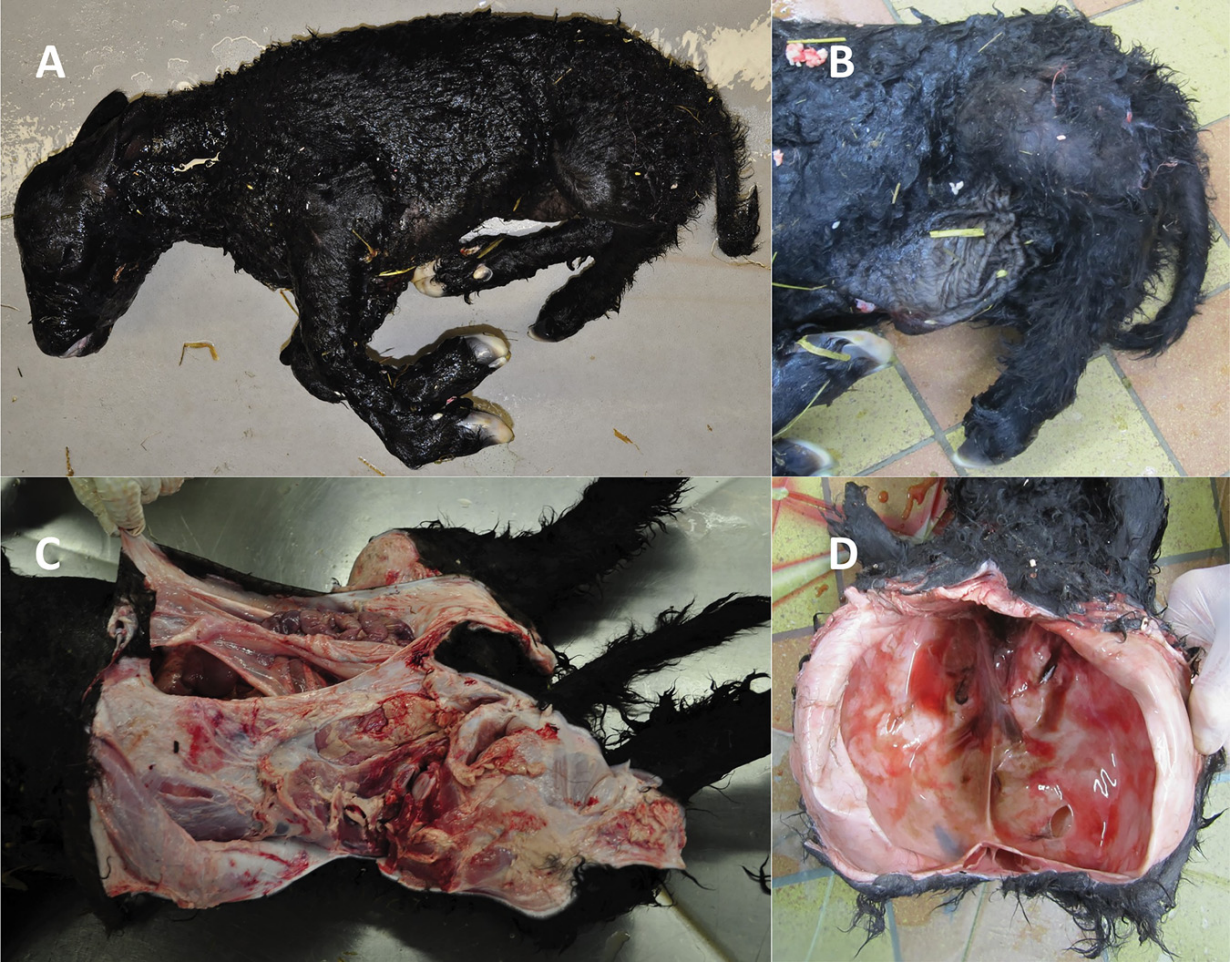
Congenital anomalies and malformations in Black Galloway calves. Stillborn calves showed several malformations including A) arthrogryposis multiplex, B) tibial hemimelia, B/C) abdominal hernia (omphalocele), and D) acerebral macrocephaly with cranioschisis. Calf V.1 (Fig 3) is shown in A, B, C and calf V.2 (Fig 3) is shown in D.

In a Black Galloway cattle family problematic pregnancies of two dams (954, 955) with signs of premature calving approx. 10–14 day before the calculated date were reported to the farm veterinarian. The veterinary medical examination revealed that both calves were not alive anymore. It was possible to deliver one calf (V.1) vaginally after manual correction of leg positions. The other calf (V.2) had an abnormal posture that could not be corrected and therefore had to be extracted by fetotomy. Stillbirth was confirmed in both calves by fetal atelectasis of the lungs. Hence, extramedullar haematopoesis was found as an additional fetal character. Both calves showed multiple malformations, *i*.*e*. abdominal hernia, arthrogryposis multiplex, and syndromic acerebral macrocephaly (Fig 1).

**Fig 2:**
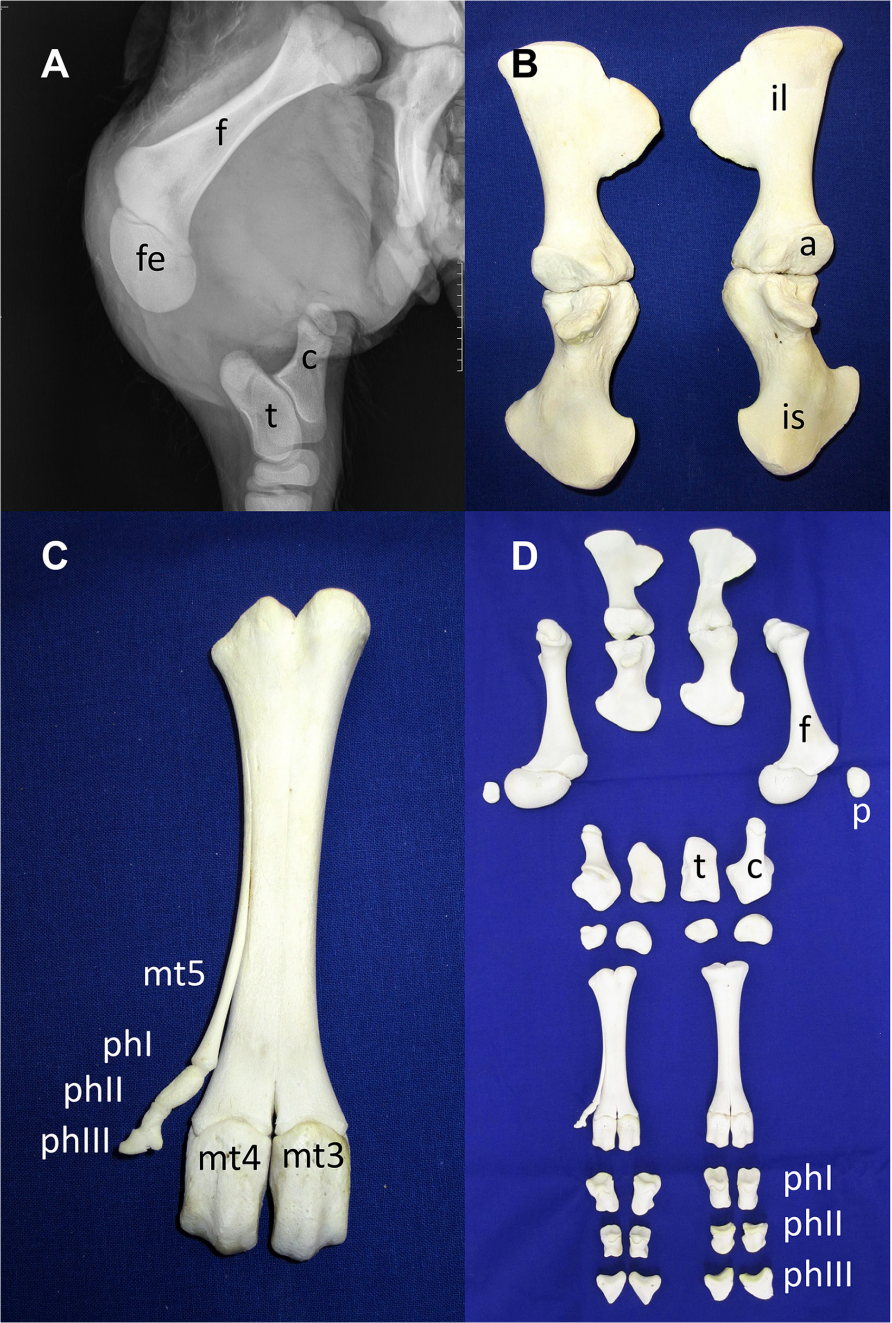
X-ray analysis and maceration of the hind legs of affected calf V.1. X-ray analysis showed complete abscence of the tibia and fibular rudiment. Visible bone rudiments represent rounded distal femoral epiphysis and malformed femoral condyles (A, fe). Maceration of the hind legs revealed separated and disconnected pelvic bones (B, D) and polydactyly of the metatarsus with rudimentary phalangeal bones (C mt5, phI-III, D). f: Femur; fe: Distal femoral epiphysis; c: Calcaneus; t: Talus; mt3-5: Metatarsals 3–5; phI-III: Phalanges I-III; il: Ilium; a: Acetabulum; is: Ischium; p: Patella.

An X-ray analysis and maceration of the hind legs of calf V.1 revealed a specific aplasia of the tibia (Fig 2). Further malformations were apparent affecting the pelvic bone. The left and right parts of the pelvic bone were not connected due to the lack of the pubic bone. The distal epiphysis of the femur was enlarged and rounded and did not show a normal development of the condyles and intercondylar fossa (Fig 2A, fe). Further distal, a rudimentary lateral metatarsal fifth phalanx can be seen (preaxial polydactyly) (Fig 2C, mt5). A detailed anatomical and pathological examination of calf V.2 was difficult due to the necessary veterinary removal and fragmentation of the fetus by fetotomy.

To exclude that the malformations were caused by infections of the mothers during pregnancy and/or calves a detailed microbiological analysis of common protozoa, bacteria and viruses, *i*.*e*. Neospora canium, Salmonella spp., Brucella spp., Leptospira spp., Mycobacterium avium subsp. paratuberculosis, Coxiella burnetii, Chlamydia spp., Schmallenberg virus, Bovine Herpesvirus, and Pestivirus, was performed. No antigens or antibodies were detected (data not shown) and therefore an infectious cause was considered unlikely.

### Sequence analysis of the *ALX4* gene

**Fig 3:**
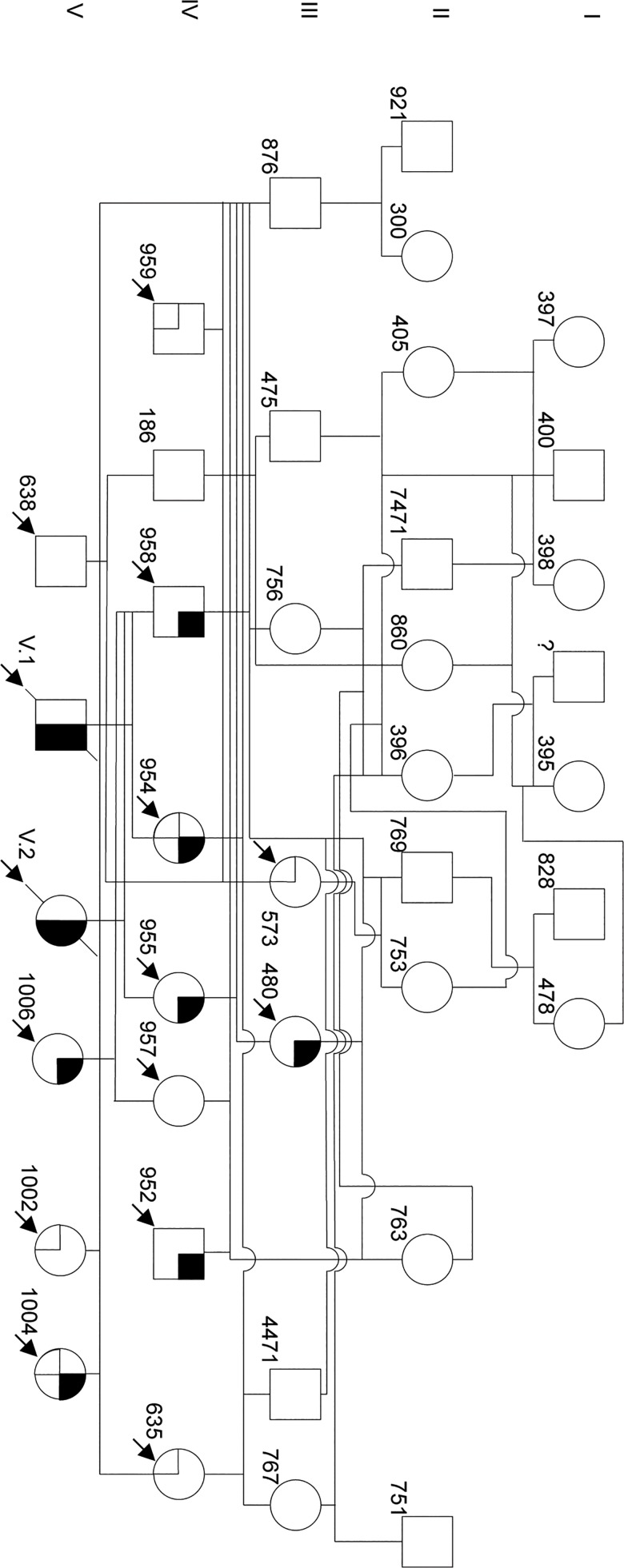
Pedigree analysis of the affected Galloway cattle family. The graph shows the 5 generation pedigree of the tibial hemimelia affected Galloway cattle. Pedigree symbols are according to the standardized human pedigree nomenclature [58]. Numbers at the lower left of the symbols refer to the 3 or 4 last digits of the individual ear tag numbers. The affected calves (V.1, V.2) did not receive an ear tag number due to stillbirth. Arrows indicate samples that were provided for analysis. Symbol segments indicate the presence of the exon 2 (black) and exon 4 (cross hatched) duplications. The pedigree was drawn using Microsoft PowerPoint for Mac 2011 Version 14.4.8 (150116).

**Fig 4:**
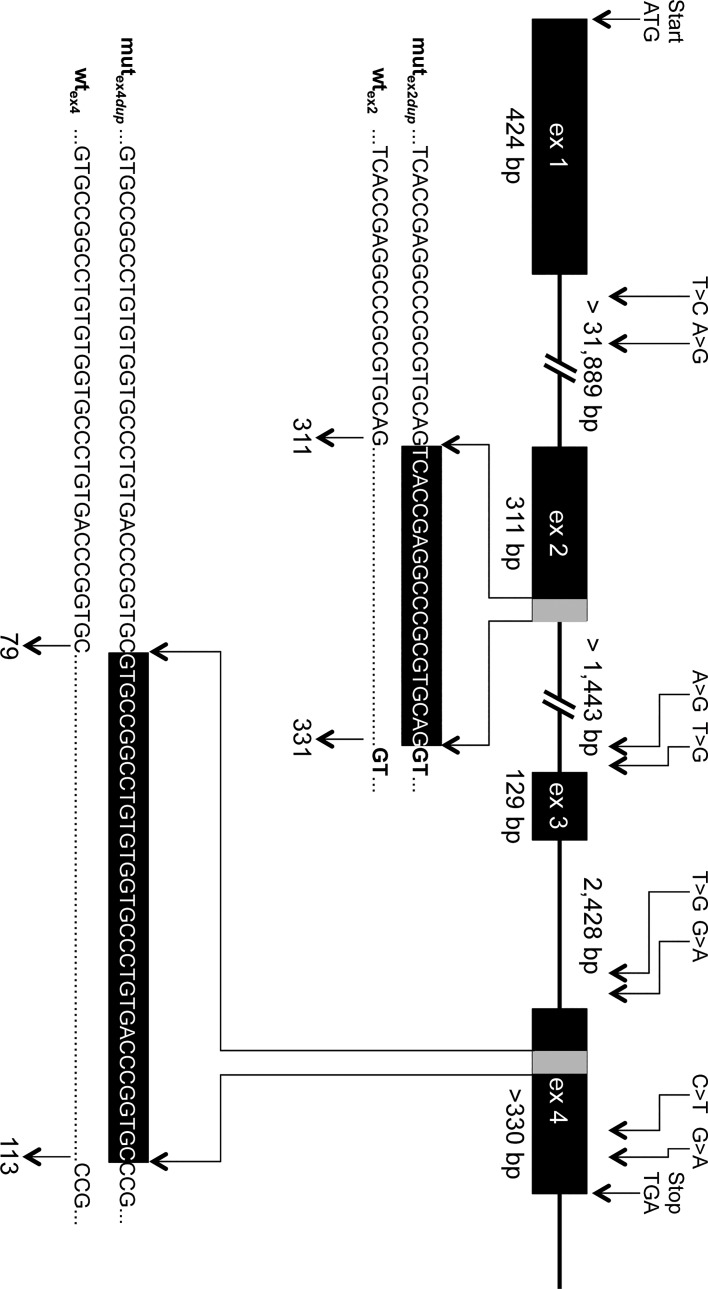
Genomic structure of the bovine *ALX4* gene and positions of variants detected in Galloway cattle. The genomic structure of the bovine *ALX4* gene as deduced from AC_00172 (Bos_taurus_UMD_3.1), NC_007313 (Btau_4.6.1), and NM_001030304 is depicted [33]. Sizes of intron 1 and 2 are not yet known due to larger gaps. Numbering of positions refers to AC_000172. Duplicated sequences in exon 2 and exon 4 in the affected animals are shown with gray bars. Numbers below the sequences indicate the corresponding nucleotide positions of the wildtype (wt_ex2_, wt_ex4_) and mutated (mut_ex2dup_, mut_ex4dup_) alleles within the corresponding exon. Positions of the SNPs and duplications according to HGVS [35] are listed in Table 2.

Tissue samples of the two calves and 13 relatives of the Galloway pedigree (Fig 3) were made available by the Galloway breeder for further molecular genetic examination. From the pedigree shown in Fig 3 an autosomal recessive inheritance of the defect can be predicted. DNA sequence comparison of the coding and flanking intronic regions of the *ALX4* gene in seven animals of the Galloway cattle family revealed 10 SNPs (Fig 4, Table 2). Four of the SNPs detected in introns were located close to the splice acceptor sites of exon 3 and 4 (Table 2). Although these SNPs were rather distant from the exon-intron boundaries with 18 bp (g.75363920T>G), 24 bp (g.75361369G>A), 25 bp g.75363927A>G), and 84 bp (g.75361429T>G), the effect on splicing was analyzed *in silico* by using different splice prediction algorithms recommended by the unclassified genetic variants guidelines [41]. Only SNP g.75363920T>G located in the splice acceptor region 18 bp upstream of exon 3 resulted in a marginal increase of the prediction scores from 0.96 (wild type) to 0.97 (mutant) when using NNSPLICE 0.9 [40].

**Table 2:** Polymorphisms detected in the bovine *ALX4* gene of Galloway cattle^a)^.

Description^b)^	Location	Type	Effect
g.75178686A>G	intron 1	SNP	
g.75178722T>C	intron 1	SNP	
c.75154399_75154418dup	exon 2	duplication	frameshift
g.75363920T>G	intron 2	SNP	
g.75363927A>G	intron 2	SNP	
c.75363774C>T	exon 3	SNP	synonymous
c.75363861T>C	exon 3	SNP	synonymous
g.75361369G>A	intron 3	SNP	
g.75361429T>G	intron 3	SNP	
c.75361028G>A	exon 4	SNP	synonymous
c.75361094C>T	exon 4	SNP	synonymous
c.75361268_75361301dup	exon 4	duplication	frameshift

*Note*. a) Polymorphisms were identified by sequence comparison of animals 573, 954, 955, 957, 958, V.1, V.2 (Fig 3)

b) Nomenclature according to HGVS refers to AC_000172 (Bos_taurus_UMD3.1) [35]. Note that sequence variations are listed according to their location.

**Fig 5:**
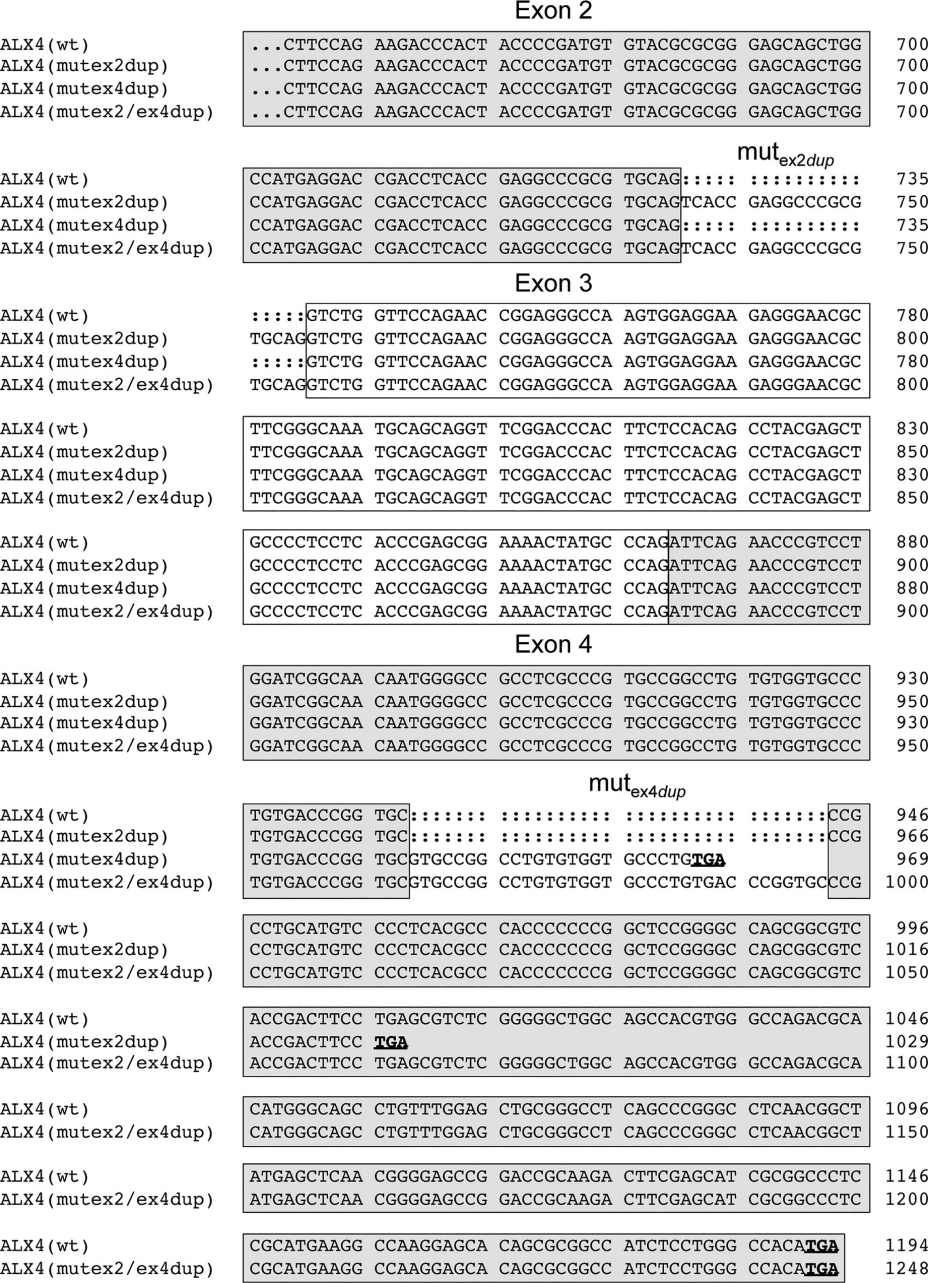
Alignment of coding sequences of *ALX4* variants. The coding sequences of the four *ALX4* variants beginning in exon 2 are shown. The exonic regions are indicated with boxes. Stop codons are shown in bold and are underlined. Numbering refers to the respective nucleotide position within the variant.

**Fig 6:**
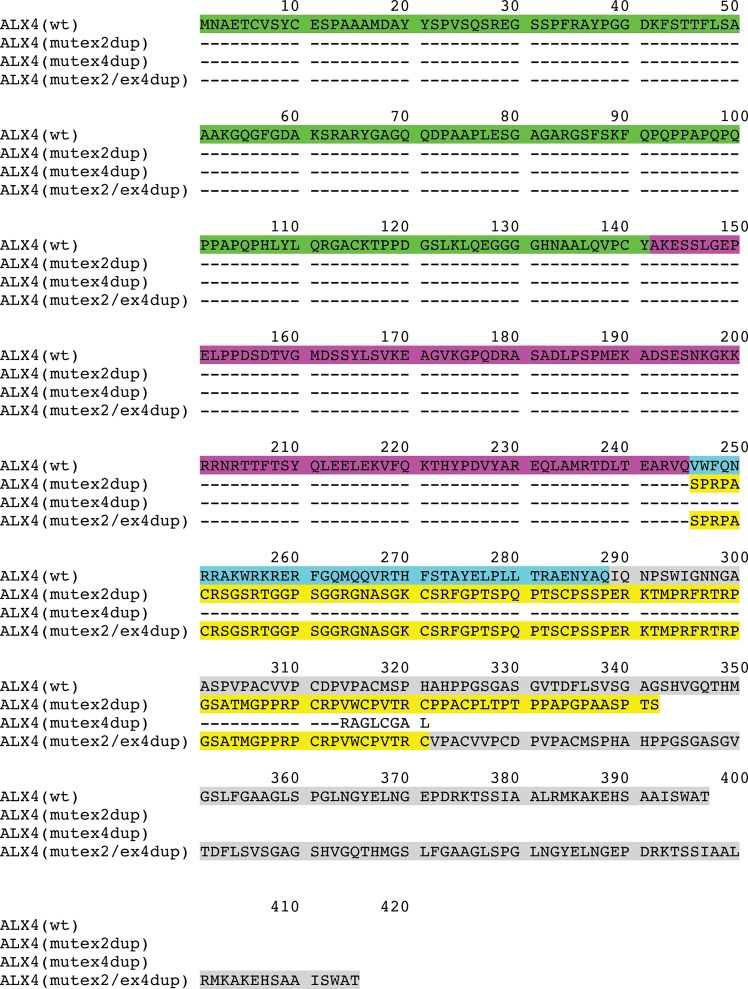
Deduced amino acid sequences of *ALX4* variants. Amino acid sequences were deduced from the coding sequences of the *ALX4* variants. Dashes indicate identical amino acid sequences. Corresponding exonic regions are indicted with different colours. Amino acids encoded by exon 1 are shown in green, exon 2 in magenta, exon 3 in cyan, and exon 4 in grey. The altered amino acid sequence due to the exon 2 duplication is shown in yellow. The truncated amino acid sequence of the ALX4(mut_ex4dup_)-variant is shown in plain text. Wt: Wild type.

The SNPs in the coding regions were synonymous mutations. In exon 2 and exon 4 duplications of 20 bp and 34 bp were detected in the affected animals and their parents. Both duplications result in a frameshift leading to a premature stop codon and truncated protein. Fig 5 shows the cDNA alignments of the different *ALX4* variants. The resulting proteins are depicted in Fig 6. The affected calfs were homozygous only for the duplication in exon 2 (Table 3). Both cattle were paternal half-siblings of sire #958, who was heterozygous for the duplication in exon 2. Mother #954 of V.1 was carrying both duplications and mother #955 (of V.2) was heterozygous for the exon 2 duplication only. Her mother #480 was also heterozygous for the duplication in exon 2.

**Table 3:** *ALX4* genotypes of animals of the Galloway cattle family.

Animal ID	exon 2	exon 4	Phenotype^a)^	F^b)^	M^c)^
V.1	mut_ex2dup_/mut_ex2dup_	wt_ex4_/wt_ex4_	THA	958	954
V.2	mut_ex2dup_/mut_ex2dup_	wt_ex4_/wt_ex4_	THA	958	955
480	mut_ex2dup_/wt_ex2_	wt_ex4_/wt_ex4_	THC	769	763
952	mut_ex2dup_/wt_ex2_	wt_ex4_/wt_ex4_	THC	876	763
954	mut_ex2dup_/wt_ex2_	mut_ex4dup_/wt_ex4_	THC	876	573
955	mut_ex2dup_/wt_ex2_	wt_ex4_/wt_ex4_	THC	876	480
958	mut_ex2dup_/wt_ex2_	wt_ex4_/wt_ex4_	THC	876	756
1004	mut_ex2dup_/wt_ex2_	mut_ex4dup_/wt_ex4_	THC	876	635
1006	mut_ex2dup_/wt_ex2_	wt_ex4_/wt_ex4_	THC	958	957
573	wt_ex2_/wt_ex2_	mut_ex4dup_/wt_ex4_	THF	769	753
635	wt_ex2_/wt_ex2_	mut_ex4dup_/wt_ex4_	THF	4471	767
638	wt_ex2_/wt_ex2_	wt_ex4_/wt_ex4_	THF	186	573
957	wt_ex2_/wt_ex2_	wt_ex4_/wt_ex4_	THF	876	763
959	wt_ex2_/wt_ex2_	mut_ex4dup_/wt_ex4_	THF	876	573
1002	wt_ex2_/wt_ex2_	mut_ex4dup_/wt_ex4_	THF	876	635

*Note*. a) THA: Tibial hemimelia affected; THC: Tibial hemimelia carrier; THF: Tibial hemimelia free

b) F: Father

c) M: Mother (see also Fig 3).

To determine the frequency of both duplications in German Galloway cattle (GA: Black, Red, Belted, Riggit Galloway; WGA: White Galloway) and other cattle breeds, we screened randomly selected cattle of GA, WGA, HF and 21 different breeds (see Materials and Methods). The duplications were only detected in Galloway cattle (Table 4). In Black/Red/Belted/Riggit Galloway the exon 2 duplication allele frequency was 0.01 and the exon 4 duplication allele frequency was 0.23. WGA showed higher frequencies for both duplications with 0.06 and 0.38, respectively.

**Table 4:** Prevalence of *ALX4* gene duplications in exon 2 and exon 4 in different cattle breeds.

Breed		Exon 2			Exon 4		N
	wt/wt	wt/dup	dup/dup	wt/wt	wt/dup	dup/dup	
GA	1641	47	0	1026	561	101	1688
WGA	252	37	0	110	137	42	289
HF	876	0	0	876	0	0	876
AA	2	0	0	2	0	0	2
AU	1	0	0	1	0	0	1
BA	5	0	0	5	0	0	5
BS	3	0	0	3	0	0	3
CHA	9	0	0	9	0	0	9
CHI	7	0	0	7	0	0	7
GAN	5	0	0	5	0	0	5
GB	2	0	0	2	0	0	2
GS	5	0	0	5	0	0	5
GL	5	0	0	5	0	0	5
GY	1	0	0	1	0	0	1
HE	4	0	0	4	0	0	4
SH	3	0	0	3	0	0	3
LI	4	0	0	4	0	0	4
PI	7	0	0	7	0	0	7
RH	8	0	0	8	0	0	8
GRH	1	0	0	1	0	0	1
AN	3	0	0	3	0	0	3
WB	2	0	0	2	0	0	2
BB	2	0	0	2	0	0	2
WP	7	0	0	7	0	0	7

*Note*. GA: Black/Red/Belted/Riggit Galloway; WGA: White Galloway; HF: Holstein Friesian; AA: Aberdeen Angus; AU: Aubrac; BA: Blonde d´Aquitaine; BS: Brown Swiss; CHA: Charolais; CHI: Chianina; GE: German Angus; GB: German Black Pied cattle; GS: German Simmental; GL: Glanrind; GY: German Yellow Cattle; HE: Hereford; SH: Scottish Highland; LI: Limousin; PI: Piemonteser; RH: Red Holstein; GRH: German Red Highlander; AN: Angler; WB: Welsh Black; BB: Belgian Blue; WP: White Park

## Discussion

In humans tibial hemimelia or agenesis has been described in combination with other more apparent defects, *e*.*g*. ectrodactyly and femoral duplication in Gollop-Wolfgang complex [42–44], multiple exostoses and mental retardation in Langer-Giedion syndrome [45], or partial alopecia, frontonasal dysplasia, and hypogonadism in Potocki-Shaffer syndrome [46]. In Gollop-Wolfgang complex and Langer-Giedion syndrome, deletions on chromosome 8q have been identified, whereas Potocki-Shaffer syndrome is caused by deletion and/or mutations of the *ALX4* gene on chromosome 11p11.2 [7, 17]. Thus, human tibial hemimelia seems to be part of a group of heterogeneous genetic disorders.

Pathological findings more precisely reflecting the malformations found in the two Galloway calves have been described in the naturally occurring murine *Alx4Lst* mutant (Strong´s luxoid) with defects of the ventral body wall and pelvic girdle together with polydactyly [10, 47]. In addition, a study in the ENU-induced mouse mutant *Alx4* ^*m1Yzcm*^ described multiple abnormalities including preaxial polydactyly, malformation (truncation) of the tibia, loss of pubic bones, and formation of omphalocele [48]. These findings show certain similarities to the phenotypic characteristics of the two stillborn calves reported herein and support our hypothesis that *ALX4* is the most likely candidate for TH in Galloway cattle. According to the morphological groups in humans, the two affected calves represented type 3 of congenital tibial aplasia lacking the proximal part of the tibia [32]. A similar pathology has been reported in Shorthorn cattle, however, there is no scientific publication available on the genetic cause in this breed [25]. Apart from that, a DNA-based test in Shorthorn cattle is commercially available based on the US patent US 8,158,356 B2 [49], claiming a large deletion of more than 129 kb of BTA15q (75.18–75.31Mb) including exon 1 of the bovine *ALX4* gene. Since this deletion differs from the mutation identified here, the test for Shorthorn is not applicable in Galloway cattle.

So far only single cases of TH in Galloway, Shorthorn or Bunaji cattle have been reported and therefore the frequency of the disorder seems to be rather low [25–28]. As the defect has already been reported in the early 1950´s in Galloway cattle, it is unclear why this has not become a more substantial problem since then [26, 28, 50]. A possible explanation for this discrepancy could be a high number of unreported cases of TH. On the other hand, already in 1979 a strategy for controlling tibial hemimelia syndrome in Galloway cattle has been published [50]. This approach included controlled matings with pregnancy termination at day 90 and subsequent pathological investigation of the fetuses. Today, transrectal ultrasonography seems to be a practical tool to examine fetuses for complex malformations during pregnancy [51].

While none of the two duplications within the *ALX4* gene were detected in 876 German HF and 21 other breeds, the frequency of the mutated allele in exon 2 of the sample of Galloway cattle analyzed here, was relatively high with 1%. 47 heterozygous animals for the exon 2 duplication were identifided within the 1688 animals genotyped. Regarding the exon 2 duplication the analyzed population was in Hardy-Weinberg equilibrium (Chi2 = 0.34). This is in contrast to the exon 4 duplication, where 561 heterozygous and 101 homozygous animals were identified resulting in a significant departure from HWE with a Chi2-value of 4.23. It is unclear why there was a significant higher number of observed homozygous exon 4 duplication carriers, especially as this mutation has no effect on the development of TH. Although, there is no scientific evidence available for Galloway cattle, it could be speculated that the heterozgyous duplication in exon 2 together with the homo- or heterozygous exon 4 duplication may result in a diserable phenotype and therefore lead to an increased allele frequency. This assumption is supported by the observation that heterozygous carriers of the *ALX4* deletion in Shorthorn cattle are preferred sires due to their straight hind limbs and long shaggy hair coat [29]. Consequently, more than half of the top ten Shorthorn sires were putative carriers in 2004. In the White Galloways cattle analyzed here both duplications were in Hardy-Weinberg equilibrium.

To further analyze the different effects of the two duplications on the function of ALX4, we performed an *in silico* protein structure prediction with SWISS-Model software [52, 53]. As the solution NMR structure of the homeodomain of the human ALX4 protein has been determined recently, this can be used for homology modelling [54, 55]. The bovine wild type *ALX4* gene codes for a protein of 397 amino acids, whereas the duplication in exon 2 would result in a truncation of 342 amino acids and the duplication in exon 4 of 322 amino acids. When both duplications are present a protein of 415 amino acids would be expected. While the truncated ALX4 protein resulting from the exon 2 duplication shares only 61.7% N-terminal amino acids with the wild type, the ALX4 protein with the exon 4 duplication is still 79.1% homologous. Although the protein with both duplications is 18 amino acids longer than the wild type ALX4, it shares also only the same 245 N-terminal amino acids as the mut_ex2dup_-variant.

**Fig 7:**
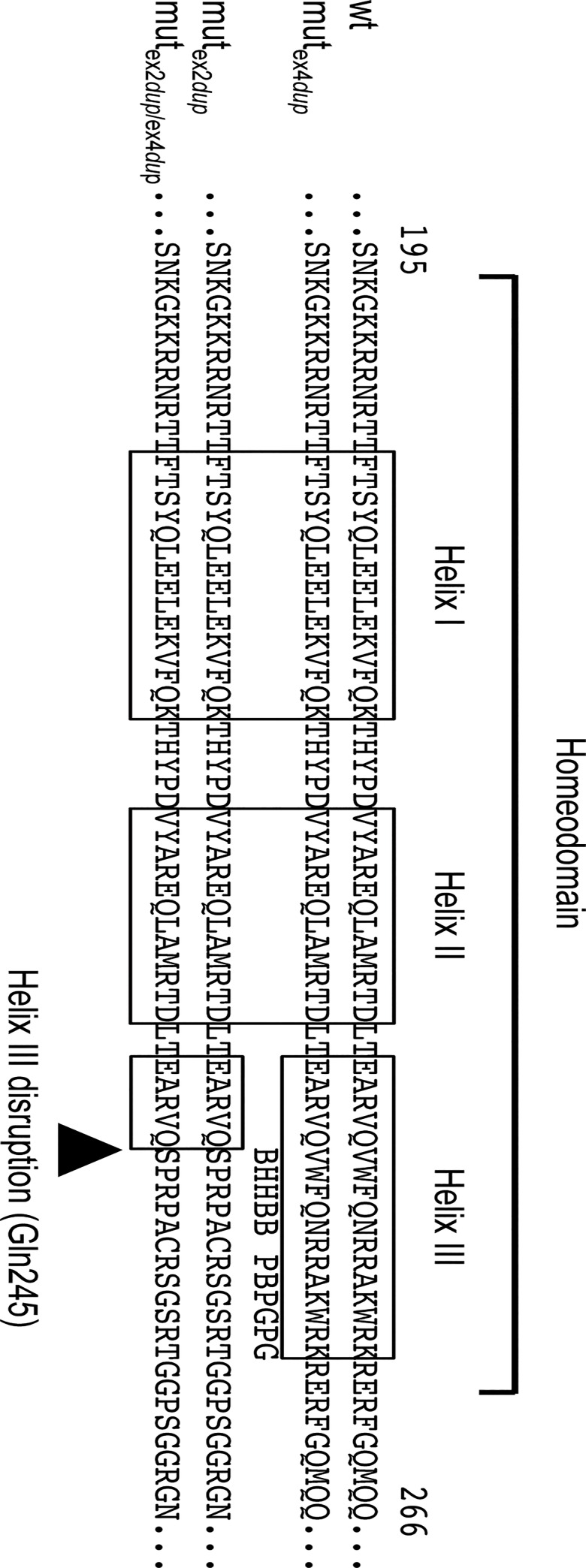
Comparison of the bovine ALX4 homeodomains of the exon 2 and exon 4 duplication variants. Alignment of the predicted mut_ex2dup_, mut_ex4dup_, and mut_ex2dup/ex4dup_ ALX4 proteins. The location of the homeodomain consensus regions are indicated with open boxes. The highlighted amino acid positions are highly conserved functional residues in helix III. B: base contact site; G: paralog-group residue; H: hydrophobic core site; P: phosphate backbone contact site [59].

**Fig 8:**
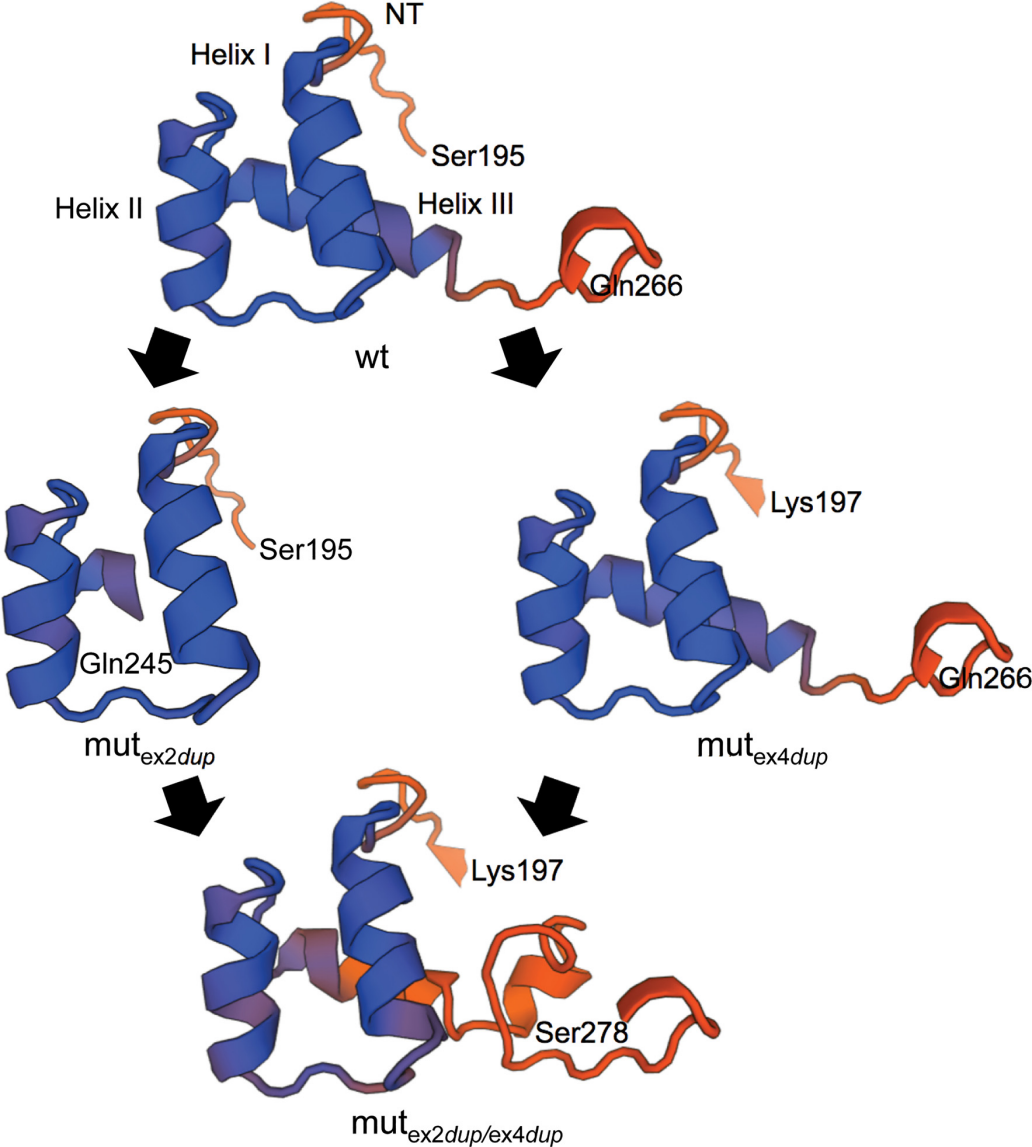
Homology modelling of the homeodomains of the exon 2 and exon 4 duplication ALX4 protein. Protein structures were predicted using the SWISS-Model workspace [52, 53]. The human ALX4 homeodomain structure has been determined by solution NMR and was used for homology modelling [54]. The three homeodomain helices (helix I, helix II, helix III) and amino acids with corresponding locations are indicated. NT: N-terminal arm.

At the C-terminal end of ALX4 an OAR domain is located between positions 377 to 390 [56]. This domain is absent in both the exon 2 and exon 4 duplication. Hence, the loss of the OAR domain cannot explain the development of TH only in the animals homozygous for the exon 2 duplication. However, due to the duplication in exon 2, important parts of helix III of the homeodomain which is necessary for DNA binding, are disrupted [57]. Whereas, in the exon 4 duplication variant the homeodomain is completely preserved. Fig 7 shows the protein alignments of the three variants and the location of the important DNA binding helices of the homeodomain. Consequently, the effect of the exon 2 duplication on the secondary or tertiary structure of the homeodomain was analyzed. Fig 8 shows the results of the protein homology modelling analysis of the homeodomains of the three bovine ALX4 variants in comparison to wild type ALX4 based on the solution NMR structure of the human ALX4 homeodomain [54, 55]. As expected, the exon 2 duplication resulted in a subtantially altered secondary and tertiary structure of the homeodomain which supports the explaination for a loss of function. Although both duplications result in a longer protein, the addition of the exon 4 duplication does not restore the ALX4 homeodomain structure.

In conclusion, TH in Galloway cattle is most likely caused by a duplication of 20 bp in exon 2 of the *ALX4* gene resulting in a frameshift and disruption of helix III of the homeodomain. As the candidate causal defect for TH in Galloway cattle has now been resolved, it will be possible to test the population and/or important breeding animals and implement genotyping results into breeding programs to avoid a further uncontrolled spread of the recessive mutant allele.
