# Correction: A 20 bp Duplication in Exon 2 of the Aristaless-Like Homeobox 4 Gene (*ALX4*) Is the Candidate Causative Mutation for Tibial Hemimelia Syndrome in Galloway Cattle

**DOI:** 10.1371/journal.pone.0310790

**Published:** 2024-09-19

**Authors:** Bertram Brenig, Ekkehard Schütz, Michael Hardt, Petra Scheuermann, Markus Freick

After publication of this article [1], the authors discovered that the position of the 20 bp duplication in exon 2 of the bovine *ALX4* gene was not correctly depicted in Fig 4. With the updated annotation of the *ALX4* gene, the duplication is located 3 base pairs upstream at position 309 to 328 of the exon. The 3 bp shift also influences the depiction of the cDNA in Fig 5 and the amino acids at the exon boundaries in Figs 6, 7, and 8. The update of the position of the 20 bp duplication has no effect on the results and conclusions.

Here, the authors have provided updated Figs 4, 5, 6, 7, and 8 with the correct position of the 20 bp duplication in exon 2 of the bovine *ALX4* gene.

**Fig 4:**
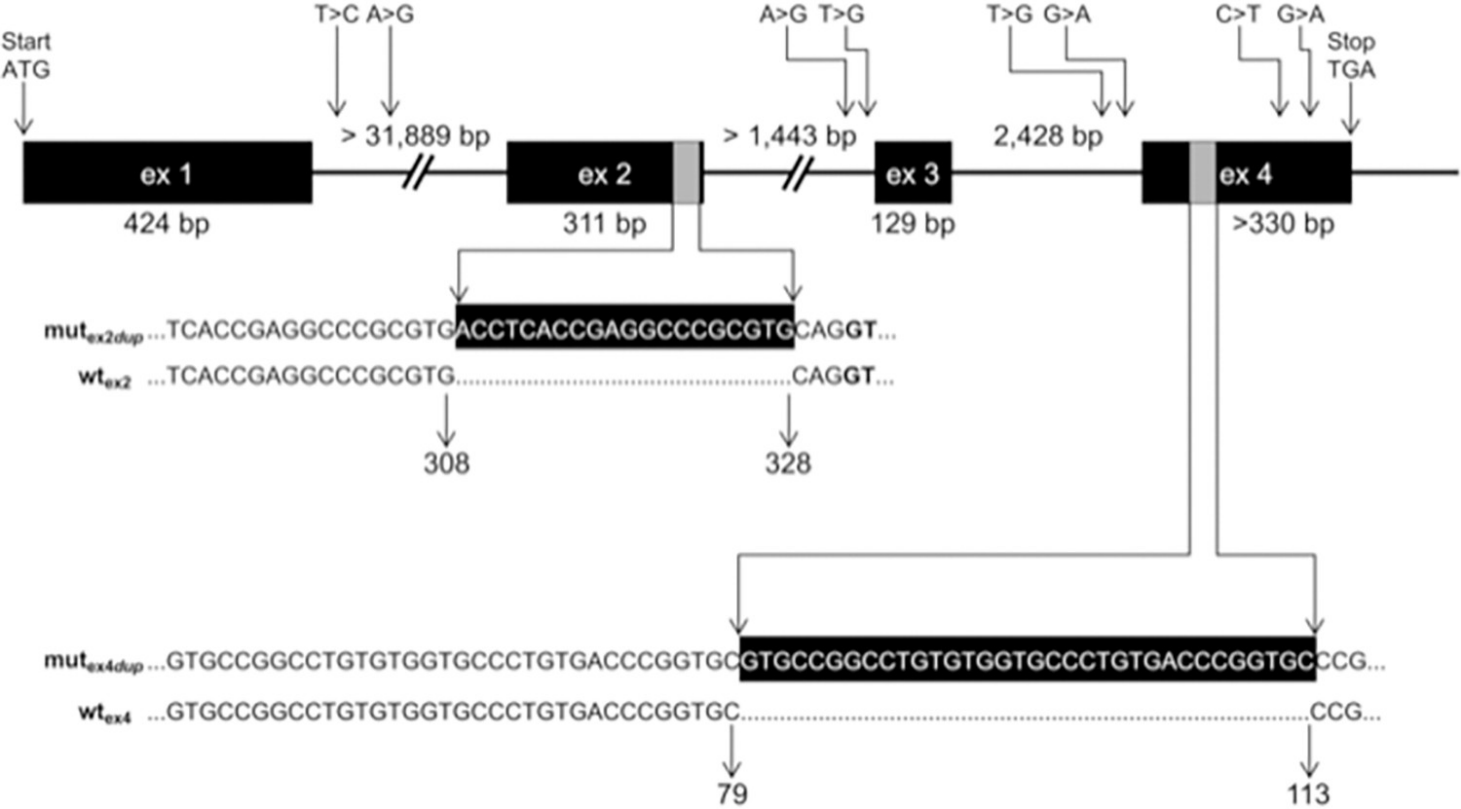
Genomic structure of the bovine *ALX4* gene and positions of variants detected in Galloway cattle. The genomic structure of the bovine *ALX4* gene as deduced from AC_00172 (Bos_taurus_UMD_3.1), NC_007313 (Btau_4.6.1), and NM_001030304 is depicted [33]. Sizes of intron 1 and 2 are not yet known due to larger gaps. Numbering of positions refers to AC_000172. Duplicated sequences in exon 2 and exon 4 in the affected animals are shown with gray bars. Numbers below the sequences indicate the corresponding nucleotide positions of the wildtype (wt_ex2_, wt_ex4_) and mutated (mut_ex2dup_, mut_ex4dup_) alleles within the corresponding exon. Positions of the SNPs and duplications according to HGVS [35] are listed in Table 2.

**Fig 5:**
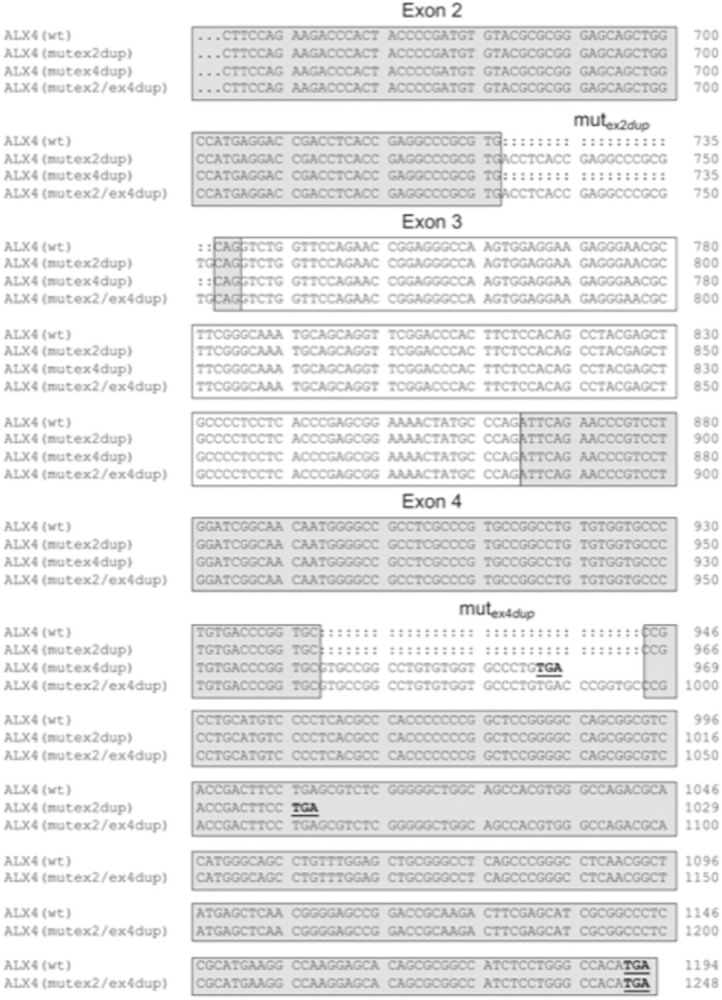
Alignment of coding sequences of *ALX4* variants. The coding sequences of the four *ALX4* variants beginning in exon 2 are shown. The exonic regions are indicated with boxes. Stop codons are shown in bold and are underlined. Numbering refers to the respective nucleotide position within the variant.

**Fig 6:**
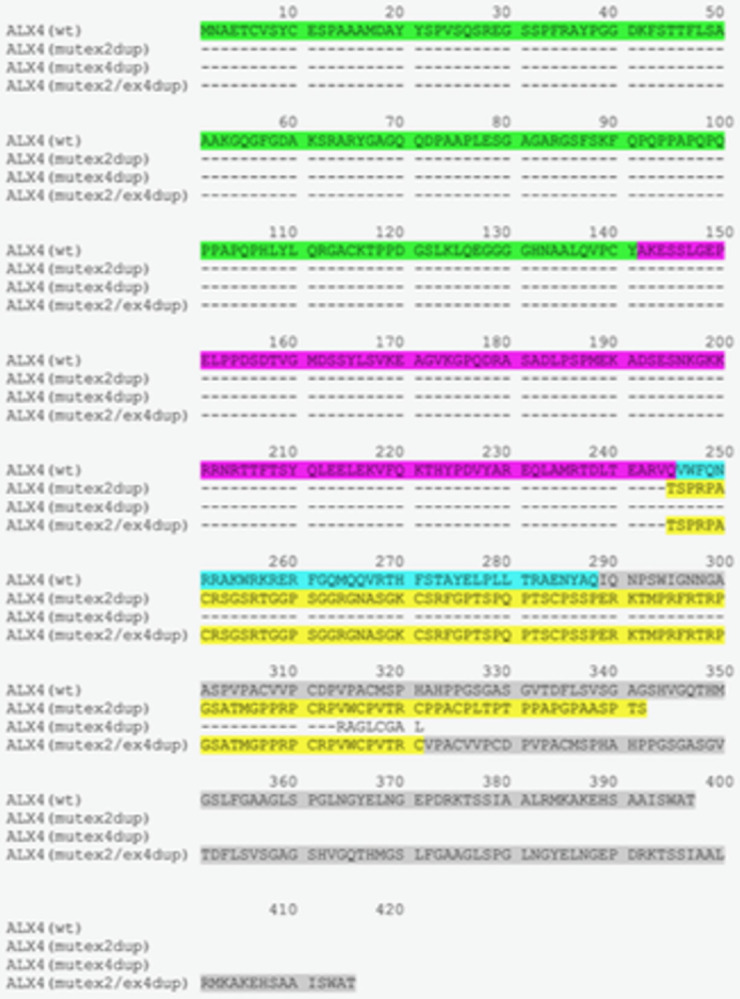
Deduced amino acid sequences of *ALX4* variants. Amino acid sequences were deduced from the coding sequences of the *ALX4* variants. Dashes indicate identical amino acid sequences. Corresponding exonic regions are indicted with different colours. Amino acids encoded by exon 1 are shown in green, exon 2 in magenta, exon 3 in cyan, and exon 4 in grey. The altered amino acid sequence due to the exon 2 duplication is shown in yellow. The truncated amino acid sequence of the ALX4(mut_ex4dup_)-variant is shown in plain text. Wt: Wild type.

**Fig 7:**
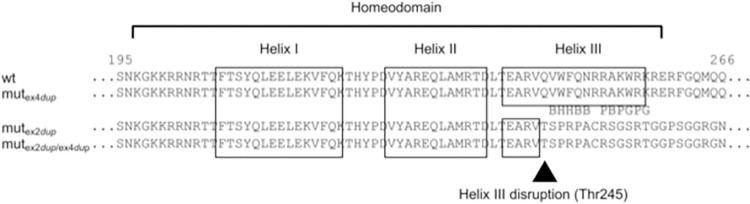
Comparison of the bovine ALX4 homeodomains of the exon 2 and exon 4 duplication variants. Alignment of the predicted mut_ex2dup_, mut_ex4dup_, and mut_ex2dup/ex4dup_ ALX4 proteins. The location of the homeodomain consensus regions are indicated with open boxes. The highlighted amino acid positions are highly conserved functional residues in helix III. B: base contact site; G: paralog-group residue; H: hydrophobic core site; P: phosphate backbone contact site 59].

**Fig 8:**
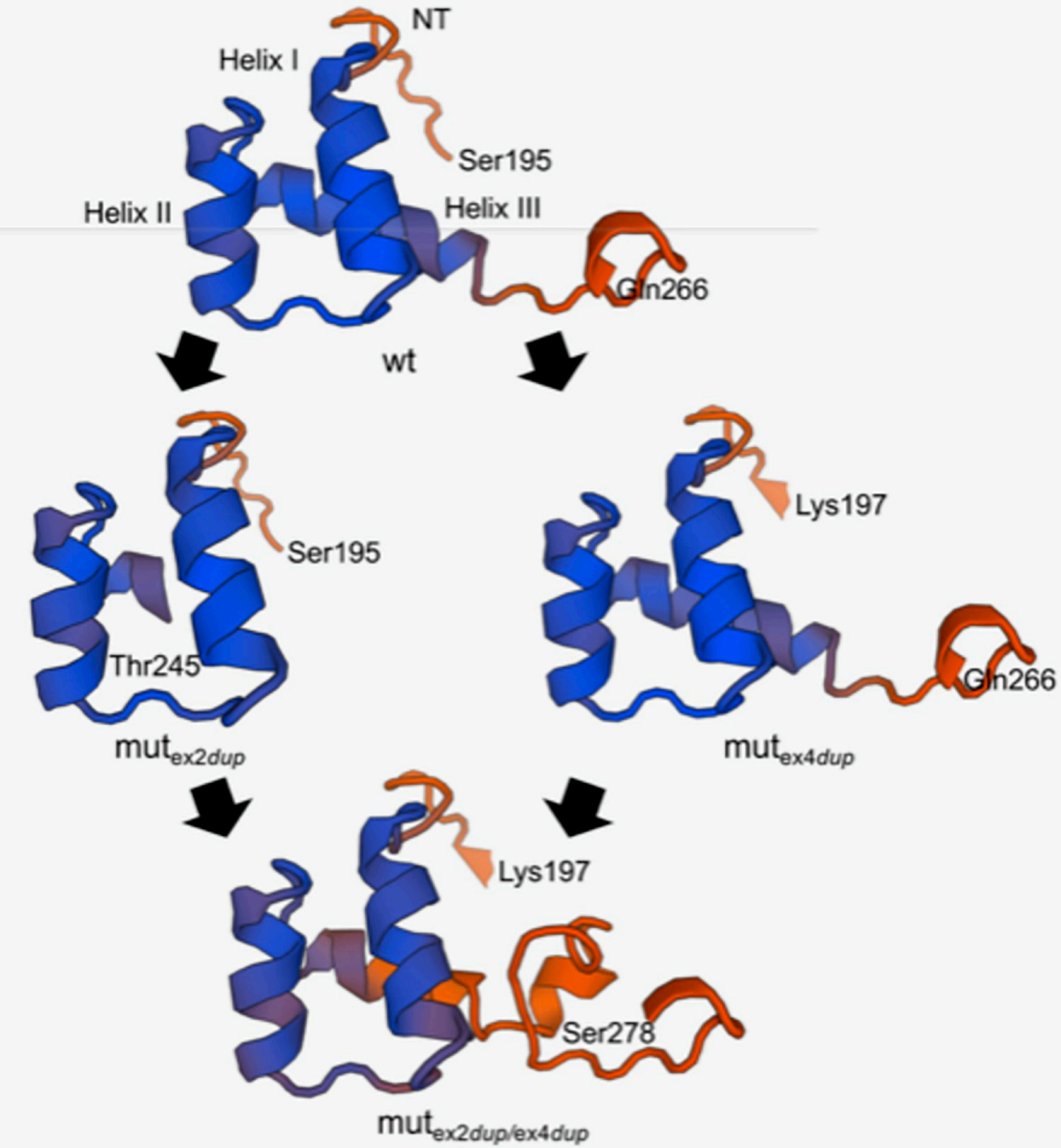
Homology modelling of the homeodomains of the exon 2 and exon 4 duplication ALX4 protein. Protein structures were predicted using the SWISS-Model workspace 52, 53]. The human ALX4 homeodomain structure has been determined by solution NMR and was used for homology modelling 54]. The three homeodomain helices (helix I, helix II, helix III) and amino acids with corresponding locations are indicated. NT: N-terminal arm.

## References

[1] Brenig B, Schütz E, Hardt M, Scheuermann P, Freick M (2015) A 20 bp Duplication in Exon 2 of the Aristaless-Like Homeobox 4 Gene (*ALX4*) Is the Candidate Causative Mutation for Tibial Hemimelia Syndrome in Galloway Cattle. PLoS ONE 10(6): e0129208. 10.1371/journal.pone.0129208 26076463 PMC4468193

